# Effects of caffeine intake and exercise intensity on executive and arousal vigilance

**DOI:** 10.1038/s41598-020-65197-5

**Published:** 2020-05-21

**Authors:** Carlos Sanchis, Esther Blasco, Fernando G. Luna, Juan Lupiáñez

**Affiliations:** 1 Faculty of Physical Education & Sport Sciences, Catholic University of Valencia, Valencia, 46001 Spain; 2 Department of Experimental Psychology, Mind, Brain and Behavior Research Center, University of Granada, 18071 Granada, Spain; 3 Instituto de Investigaciones Psicológicas (IIPsi, CONICET-UNC), Facultad de Psicología, Universidad Nacional de Córdoba, Córdoba, Argentina

**Keywords:** Attention, Randomized controlled trials

## Abstract

During physical efforts and sport practice, vigilance is responsible for maintaining an optimal state of activation, guaranteeing the ability to quickly respond and detect unexpected, but critical, stimuli over time. Caffeine and physical exercise are able to modulate the activation state, affecting vigilance performance. The aim of the present work was to assess the specific effects and modulations of caffeine intake and two physical intensities on vigilance components. Participants performed an attentional task (ANTI-Vea) to measure the executive and arousal components of vigilance, in six double-blinded counterbalanced sessions combining caffeine, placebo, or no-ingestion, with light vs. moderate cyclergometer exercise. Exercise at moderate intensity improved executive vigilance with faster overall reaction time (RT), without impairing error rates. Instead, caffeine intake generally improved arousal vigilance. In conclusion, caffeine and acute exercise seems to moderate executive and arousal vigilance in different ways.

## Introduction

Attention includes a set of cognitive processes highly involved in common physical efforts (e.g., walking on the street, bike-riding) and sports practice performance^1^, influencing information processing to quickly and accurately respond to the environmental demands. Vigilance, as part of the attentional networks model^2^, is responsible of maintaining the required state of activation to facilitate the functioning of the attentional system, in order to appropriately detect and quickly react to stimuli in the environment^3^. From a practical point of view, vigilance would allow us to avoid potential danger and accidents during relatively long demanding psychomotor multi-task actions as cycling to work, by responding quickly to an unexpected car movement and a stop light; or to properly react to infrequent peloton movements (e.g., decelerations, crashes) and road irregularities during a challenging five hour *Tour de France* cycling stage. Therefore, vigilance has a crucial role in maintaining optimal performance and safety during sports and daily physical efforts of certain duration. Indeed, the longer the motor task or sport activity (as in aerobic endurance sports), the more relevant the vigilance state will become in the complex interactive relations among all attentional networks (alertness, orienting, and executive control)^2^.

In the present study, we aimed at investigating how vigilance is modulated by caffeine intake and acute aerobic exercise. Importantly, vigilance has been recently considered as two dissociated components, which modulates different behavioral responses of sustained attention^4^. On the one hand, the executive component of vigilance (EV) can be defined as the ability to sustain attention for detecting rare but critical events (hits) over an extended period of time, and is commonly assessed by signal detection tasks like the Sustained Attention to Response Task (SART)^5^. In this type of tasks, the vigilance decrement (e.g., a phenomenon that refers to the loss of sustained attention across time on task) is usually reported either as a prominent loss in the sensitivity (Aʹ) to correctly discriminate the rare but critical events from the frequent non-critical (e.g., ‘noise’) ones^6^, or as a progressive increment in the response willingness towards a more conservative criterion for detecting the critical events (B″)^7^.

On the other hand, the arousal component of vigilance (AV) rather reflects the capacity to quickly react to the appearance of stimuli in the environment over long time periods, without much control over responses. In tasks like the Psychomotor Vigilance Test (PVT)^8^, the AV component is measured as the speed to stop – as fast as possible – a millisecond counter by pressing any available response key. Critically, and at difference with the EV component, the AV decrement is generally observed as an increment in the mean and variability of reaction time (RT) across time on task, a behavioral pattern that reflects a progressive slowness for reacting to stimuli^9^.

A growing number of investigations in the last decades have studied the effects of light-to-moderate exercise on attention and cognition performance, generally showing an exercise-induced pre-frontal cortex activation and an improvement of the information processing speed, measured by a reduction in RT on attentional tasks^10,11^. Yet, as cognitive resources have to cope with physical and attentional demands at the same time, consequently mediating attentional resources allocation^12^, studies analyzing how different acute exercise intensities modulate attentional networks processes during a psychomotor dual-task are of special interest.

More importantly for our research, there is some evidence showing that light exercise improves performance in an executive vigilance task like the SART, increasing the subjective feeling of alertness and causing positive neuroelectric changes^13^. In fact, it has been proposed that low-to-moderate exercise stimulates the synthesis and release of norepinephrine, thus maintaining tonic activation and preventing performance decrease across time^14^. However, only a few studies have specifically assessed executive vigilance performance during or after exercise. Furthermore, no consistent results have been observed, with studies showing either a reduction on vigilance performance during heavy loaded walking^15^ or no effects on executive vigilance measures after moderate intensity cycling^16^, failing to replicate the positive impact of a pre-task effort observed by Smit *et al*.^13^ with a light intensity exercise. Whilst the negative impact observed by Eddy *et al*.^15^ could be caused by the special constraints of carrying and balancing a load during 2 hours, it remains to be investigated whether performing different physical efforts and intensities (e.g., light-to-moderate) can benefit executive vigilance and modulate related measures (e.g., sensitivity and response bias).

Regarding arousal vigilance, limited research has shown that intense exercise (near VO_2max_) can increase posterior performance (reducing mean RT)^17^ and that an intensity-dependent beneficial effect is observed in the PVT during a range of light-to-moderate exercise intensities (from 40 to 80% VT_2_)^18^. Again, the exercise induced physiological changes related to augmented neural activation (e.g., catecholamine release, cerebral blood flow) has been proposed as the explanation for the positive modulations of arousal vigilance during exercise^19^, describing an “U” shape relationship where highest intensities could exceed the optimal level of activation^20^.

Beyond exercise induced effects, physical and attentional (at rest) performance improvements after caffeine (*1,3,7-trimetilxantine*) intake are widely accepted^21–23^. Specifically, it is well known that caffeine improves RT, error rate and vigilance at rest, both in simple and complex tasks. However, the effects of caffeine on attentional networks generally depend on task constraints (simple vs. complex), low to high dopaminergic stimulation of attentional networks associated brain regions or caffeine dosage (from 0.2 to 5.5 mg/kg) among others^22,24^. Considering that caffeine intake is a generalized practice among the physically active population (e.g., coffee consumption) and among athletes (e.g., specific caffeine products for performance enhancement), together with the fact that physical efforts are able to modulate the functioning of the attentional networks, the study of the combined effects of both variables would be of special interest for optimizing attentional performance in exercise conditions.

Caffeine intake (100–800 mg) has been consistently shown to enhance vigilance after^25–28^ and, more importantly, during acute aerobic exercise and maximal voluntary contractions, at least in its executive component (100–300 mg)^26,29^. Of special interest for our work, due to its design, Hogervorst *et al*.^29^ analyzed the effects of caffeine intake (100 mg) on executive vigilance during several Rapid Visual Information Processing Tasks, where participants made a dual-task effort for 2.5 h at 60% VO_2_max plus a time-trial at 75% VO_2_max. Caffeine intake led to faster responses, and increased perceptual sensitivity and readiness to respond compared to no-caffeine beverage ingestion (despite a vigilance decrement in both conditions). However, additional research is needed to examine the caffeine-exercise effects over arousal vigilance, to compare light-to-moderate aerobic intensities modulations of executive vigilance and to define the effect of higher caffeine intakes on both vigilance components in a dual-task (exercise-attention) context, as these factors have been shown to significantly influence vigilance performance.

Furthermore, task complexity has been shown to critically influence attentional behavioral outcomes and vigilance in relation to physical exercise, as complex tasks would allow to exploit and deplete completely brain resources^8,30^. In fact, daily physical efforts and sport practice requires of a complex interaction and functioning of cognition and attention, which is only partly described by isolated vigilance measures. In contrast, only a few of the aforementioned studies regarding attention and exercise have used tasks like the Attentional Network Test (ANT) and Attentional Network Test – Interactions (ANT-I), where all attentional networks work in combination^31–33^, and none of them has assessed executive and arousal vigilance along with the classical attentional networks. In this sense, is possible that the modulation of exercise over executive and arousal vigilance performance could be differently affected in a complex task context, due to the increased cognitive demands. In this regard, Luna *et al*.^4^ have recently developed the Attentional Networks Test for Interactions and Vigilance – executive and arousal components (ANTI-Vea), which allows to assess, at the same time, both components of vigilance, while measuring the classic attentional functions. As we stated previously, we think that the distinction between arousal and executive vigilance in a multiple attentional task could lead to a better understanding of the effects of exercise and caffeine on athletes’ attentional functioning, thus helping to the development of new and improved strategies to prevent accident related injuries or to enhance information processing.

Therefore, in the current study we investigated the effects of both caffeine intake (caffeine vs. placebo vs. control) and acute aerobic exercise (light vs. moderate) over attentional performance in general and over the vigilance components (arousal vs. executive vigilance) in particular. We used the ANTI-Vea task, which has been shown to be suitable to measure all these functions^4^, to test the following hypotheses: (a). We expected overall RT in the main task and arousal vigilance performance in particular to be positively modulated by exercise intensity and caffeine intake. (b). Regarding executive vigilance, following recent evidence we expect it to be better indexed by a change in response bias and to be enhanced by caffeine intake. (c). Additionally, based on neuroelectric findings, LC-NE paradigm and information processing theories, we expect to see a positive modulation of moderate exercise intensity, exploring an exercise-caffeine interaction, on executive vigilance. (d). Complementarily, we expect 5 mg/kg caffeine intake to reduce phasic alertness in low consumers during exercise, whereas we wanted to further explore caffeine and exercise effects attentional networks, extending previous research with ANT and ANT-I tasks.

## Methods

### Participants

Eligible participants were all young active adults, between 18 and 26 y/o, enrolled in the Sport Sciences Degree at Catholic University of Valencia “*San Vicente Mártir*”. Exclusion criteria were cardiac pathologies, diabetes, vision problems, allergies to caffeine o cellulose, and existing musculoskeletal injury. A 24 participants sample size was estimated a priori with G*Power 3.1.9 (*Universität Kiel, Germany*)^34^ to reach *d* = 0.4 effect size, *1-β* = 0.8 power, and *p* = 0.05 statistical significance for a repeated measures ANOVA. This effect size is reasonable according to a meta-analysis regarding caffeine effects on physical performance^35^ and a similar research involving caffeine, acute exercise and attentional performance^36^. 27 participants were selected to participate in the study to prevent possible sample loss. Indeed, three of them dropped out before study completion of all experimental sessions: two of them due to schedule breach and one due to an injury. Then, 24 men; (age: *M* = 22.6; *SD* = 2.9), non-habitual and low caffeine consumers (see Table 1) completed the study. All of them signed an informed consent before their inclusion in the experiment and were fully informed about the study protocol, experimental sessions, relevance of commitment, and rigorous following of pre-session indications. All experimental protocols were approved by the Catholic University of Valencia Ethics Committee (UCV/2016-2017/02). Furthermore, methods described in this study were performed following the requirements of the declaration adopted by the 18th World Medical Assembly (Helsinki, 1964), revised in Fortaleza (Brazil, 2013), and the Taipei declaration (2016).

**Table 1:** Basic sample characteristics and ventilatory values.

	*M*	Min.	Max.	*SD*
Age (years)	22.6	18.0	26.0	2.9
Weight (kg)	73.9	60.0	95.0	7.7
Habitual caffeine consumption (mg)	12.08	0.0	95	28.9
Week training (h)	7.0	2.5	15.0	3.4
Training experience (years)	5.8	1.0	20.0	5.8
VT_1_ (ml·kg)	22.2	11.3	37.5	7.1
VT_2_ (ml·kg)	32.7	21.7	49.0	7.8

*Note. M* = mean values; *SD* = standard deviation.

### ANTI-Vea

**Figure 1:**
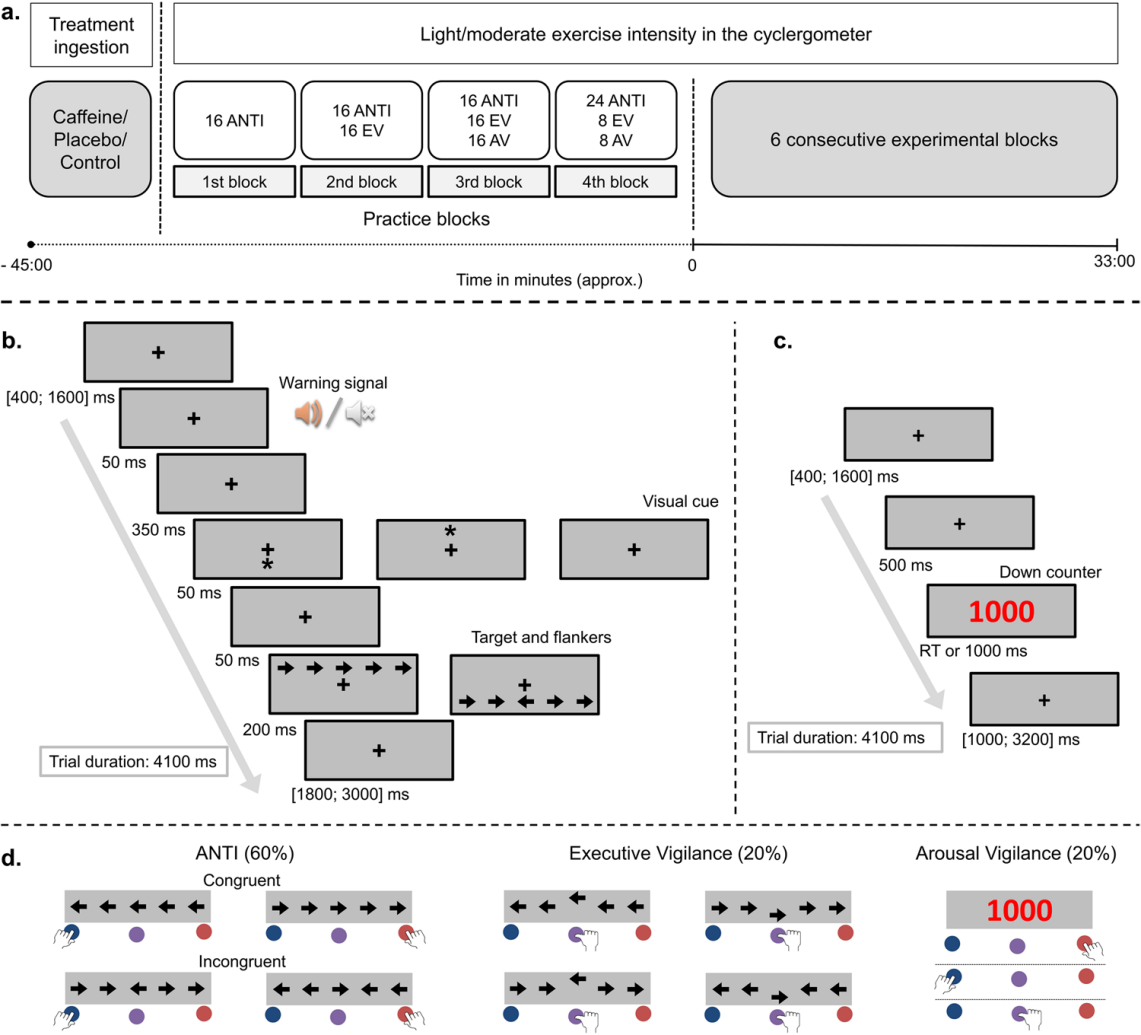
Session and experimental procedure. (**a**) Sequence timing and treatment ingestion/exercise instensity for the different sessions. In the first session, participants completed four progressive practice blocks: the 1^st^, 2^nd^, and 3^rd^ one with visual feedback, and the 4^th^ one without feedback. In the six experimental sessions, participants only completed as practice the 4^th^ block (which consist of half of one experimental block). For each practice block, the number of trials of each type is represented within the white boxes. Stimuli sequence and timing, and experimental procedure for the different type of trials are specifically represented in: (**b**) for the ANTI and EV trials, and (**c**) for the the AV trials. (**d**) Correct responses for each type of trial of the ANTI-Vea task in the current study.

This new version of the attentional networks test is suitable to assess, within a single session, the independence and interactions of the classic attentional functions (e.g., phasic alertness, orienting, and executive control), simultaneously with the decrement across time on task of the executive and the arousal vigilance components^4^. The procedure, stimuli sequence, timing, and correct responses for the three type of trials of the task (e.g., ANTI, EV, and AV) are depicted in Fig. 1. The task was completed on a laptop (*HP Pro Book, EEUU)* at approximately 60 cm from its monitor, and all stimuli were presented at eye level and in a dimly lit room. E-Prime software (Psychology Software Tools, Inc.) was used for stimuli presentation and response registration.

The ANTI trials (48 per experimental block) follow the procedure of the ANTI task, wherein participants have to perform a flanker task – which is useful to assess the executive control network – by responding to the direction the target (e.g., a central arrow) points to, while ignoring the direction pointed by the surrounding flanker arrows (e.g., two distracting arrows at each side of the target). Importantly, as shown in Fig. 1, the response stimuli: (a) could appear below or above the fixation point; (b) could be anticipated or not by a warning signal (which provides an effective measure of phasic alertness); and (c) its location on the screen regarding the fixation point could be cued either correctly, incorrectly, or not cued at all, by a visual cue (which provides a measure of attentional orienting). Therefore, the executive control effect was established as the congruency (congruent) vs. incongruency (incongruent) of the target and flankers’ arrows direction; the phasic alertness effect was defined as the presence (tone) vs. absence (no tone) of the auditive warning signal; and finally, the orienting effect was defined as the presence of a correct cued location (valid), incorrect cued location (invalid), or no spatial cue at all (no cue).

In a smaller proportion of trials (16 by block), the participants performed a typical signal detection task (similar to the SART), which is suitable to assess the EV component: they had to remain vigilant for detecting an infrequent displacement of the target (e.g., either upwards or downwards) from its central position, ignoring in these cases the direction pointed by the target (see Fig. 1) and pressing the middle button. Finally, on AV trials (16 by block, see Fig. 1) participants performed a task similar to the PVT, which is useful to obtain a direct and independent measure of the AV component: they had to stop a millisecond counter as fast as possible by pressing any available response button. Further details on stimuli properties and design can be reviewed in Luna *et al*.^4^.

### Preliminary testing

A sub-maximal incremental ramp test was performed on a cyclergometer Cardgirus Medical Pro (G&G Innovation, Álava, Spain) to obtain ventilatory thresholds (VT_1_ and VT_2_) and their associated power output. Ventilatory thresholds represent two key points of metabolic change due to increase in exercise intensity, and have been used as references for exercise intensity prescription^37^. Incremental test was automatized with 5 W/15 s steps, already used with young adults^25,29,38^. There was no warm-up and test started with 20 W. Cadence had to be maintained above 60 rpm. Heart rate (HR) was measured with a Polar RS800cx (Polar Electro, Finland) and ventilatory variables (VO_2_, VCO_2_, RER y V_E_) were registered with portable ergometer Oxycon Mobile (CareFusion, Switzerland), following manufacturer instructions for set-up and calibration. Test ended when VT_2_ was detected.

### Ventilatory thresholds detection

VT_1_ and VT_2_ were calculated automatically by the software Lab Manager v.5.31.04. Precisely, VT_1_ was calculated using V-Slope method^39^, and VT_2_ was calculated by EqCO_2_ method^40^. Associated power outputs at VT_1_ and VT_2_ were obtained and utilized to calculate 80% of VT_1_ and VT_2_.

### Exercise protocol

Experimental sessions were conducted following a constant sub-maximal exercise protocol. Participants started with a warm-up based on Schmit *et al*.^41^: 180 s at 50% of VT_2_, 120 s of progressive increase/decrease intensity until session target intensity was reached, and 90 s at session target intensity for VO_2_ stabilization. Afterwards, intensity remained constant at 80% of VT_1_ or VT_2_, with no interruptions during 33 min and 45 s. Cadence had to be maintained above 60 rpm.

### Treatment ingestion

Participants received 5 mg/kg of either anhydride caffeine, or placebo capsules with cellulose, or no-treatment at all, as control condition. Caffeine dosage was slightly greater than in previous research^31,42,43^, aiming at obtaining a clear modulation of caffeine intake. Capsules were ingested with *ad libitum* water and were identical (number, color, tact, and weight) in both treatments. In all experimental sessions, Caffeine and Placebo treatment was always administered 45 min prior attentional assessment and exercise protocol, following Huertas *et al*.^31^.

### Procedure

Participants completed eight sessions at the Catholic University of Valencia. Following previous research^31^, sessions were separated by 48–96 h to avoid the influence of exercise on attentional performance, to minimize physical or physiological changes, and to ensure caffeine removal^44^. Also following previous research^45^, instructions to restrict caffeine or stimulants ingestion 12 h before each session, vigorous physical activity during previous 24 h, and doing last meal at least 2 h before experimental session were given to all participants. In the first session, participants were familiarized with the sub-maximal incremental test and experimental instruments by completing the ANTI-Vea task while pedaling on the cyclergometer at an auto-selected intensity. Importantly, only in the first session of the study participants gradually received extensive instructions to perform correctly each type of trial of the ANTI-Vea and completed several practice blocks with visual feedback regarding incorrect responses, as in Luna *et al*. (see also Fig. 1). Next, during the second session, the sub-maximal incremental ramp test was performed to assess ventilatory thresholds and to establish, individually for each participant, the intensity of the experimental sessions. In the following six sessions, participants only completed one practice block prior to the experimental task (see Fig. 1), and were randomly assigned through a double-blinded and counterbalanced Latin-Square design to one of the following treatments: (1) 5 mg·kg Caffeine at 80% VT_1_; (2) 5 mg·kg Caffeine at 80% VT_2_; (3) Placebo at 80% VT_1_; (4) Placebo at 80% VT_2_; (5) Control at 80% VT_1_; (6) Control at 80% VT_2_. In the Caffeine and Placebo sessions, participants received the corresponding treatment and waited in the laboratory facilities. In Control sessions, they received no treatment. After 45 min, participants performed the ANTI-Vea task in the cyclergometer. To collect responses to stimuli while pedalling, three response buttons were placed in the left, middle, and right side of the cyclergometer’s handlebar. Buttons positions were adjusted individually for each participant, allowing responding with the index fingers to the left and right buttons, and with the dominant hand thumb to the middle button (see Fig. 1).

### Statistical analysis

Data analysis was performed in *Statistica Dellsoft* 12.0 (*StatSoft, Inc*.). Breath-by-breath ventilatory variables (VCO_2_, VO_2_, V_E_, EqO_2_, EqCO_2_ y RER) were analyzed for extreme and outliers’ values by box-plot method (1.5 coefficient interquartile range), recoding those values with two-sided Tukey. Then, mean values were calculated for every treatment session and participant. No HR extreme and outliers’ values were found with *Polar Protrainer* v.5 (*Polar Electro*) software. To assess exercise and treatment influence on ventilatory variables and HR, six repeated measures ANOVAs were conducted, including Exercise (Light/Moderate) and Treatment (Caffeine/Placebo/Control) as within-participant’ factors, and VCO_2_, VO_2_, V_E_, EqO_2_, EqCO_2_, RER, and HR, as dependent variables. ANTI-Vea outliers and extreme values were handled following the same procedure as Luna *et al*.^4^.

For the RT analysis (both for ANTI and EV trials), responses with RT faster than 200 ms or slower than 1500 ms (0.44%), and those with incorrect responses (7.47%), were excluded^4^. Then, two repeated measures ANOVAs were conducted, one with RT and the other with error rate (% of errors) as dependent variable, and including Phasic Alertness (No Tone/Tone), Orienting (Invalid/No Cue/Valid), Executive Control (Congruent/Incongruent), Exercise (Light/Moderate) and Treatment (Caffeine/Placebo /Control), as within-participants factors. Classic attentional networks indexes of RT and error rates performance were calculated by means’ subtraction in specific conditions, following previous studies^4^: Phasic Alertness (No Tone – Tone, only considering No Cue trials), Orienting (Invalid – Valid) and Executive Control (Incongruent – Congruent). Repeated measures ANOVAs were conducted then for each index as dependent variable, including Treatment (Caffeine/Placebo/Control) and Exercise (Light/Moderate) as within-participants factors.

Additionally, EV and AV measures were calculated per block of trials in each experimental session, in order to assess vigilance decrement across time on task. Therefore, for the analysis of EV trials, data from Phasic Alertness, Orienting and Executive Control was not considered. Following Luna *et al*.^4^, non-parametric indexes of sensitivity (A’) and response bias (B”) were calculated from Hits (e.g., correct detection on infrequent target) and False Alarms (FA, e.g., responses to the frequent stimuli with the infrequent target response)^46^. Finally, mean RT and variability (SD) of RT on Hits were also obtained. Four independent repeated measures ANOVAs were conducted, one for each dependent variable (A’, B”, mean and SD of RT), including Treatment (Caffeine/Placebo/Control), Exercise (Light/Moderate) and Block (six levels), as within-participants factors.

Furthermore, regarding AV data, mean and SD of RT, and percentage of Lapses (e.g., responses equal or higher than 600 ms) per block of trials and experimental session were calculated. The consideration of Lapses as responses with RTs equal or greater than 600 ms differs from the 500 ms threshold usually used^9^, due to the greater task demands and the higher mean RT observed in the ANTI-Vea in comparison with much simpler tasks like PVT. Then, three repeated measures ANOVAs were conducted including Treatment (Caffeine/Placebo/Control), Exercise (Light/Moderate) and Block (six levels) as within-participants factors, one for each dependent variable: mean RT, SD of RT, and percentage of Lapses. Statistical significance level and confidence intervals were set at 0.05 and 95%, respectively, for all analysis.

## Results

Participants’ basic characteristics and ventilatory values obtained in the incremental test are shown in Table 1. Mean power of Moderate and Light exercise sessions were 168.42 W (37.10 W) and 95.58 W (32.05 W), respectively.

Mean ventilatory and HR values are shown in Table 2. It was observed an important main effect of Exercise on the ventilatory values [*F* (1, 23) = 149.68, *p* < 0.001, ${\eta }_{p}^{2}$ = 0.87]. In addition, main effects of VCO_2_, VO_2_, V_E_, EqO_2_ and RER were also observed, but not for EqCO_2_ (see Table 2). Exercise × Treatment interaction was not observed for any of the physiological variables (all *F*s < 1).

**Table 2:** Mean data for each physiological variable in the Exercise conditions.

	Moderate Exercise			Light Exercise		
	Caffeine	Control	Placebo	Caffeine	Control	Placebo
VCO_2_ (ml)	2185 (92)**	2189 (79)**	2211 (89)**	1420 (72)	1403 (66)	1394 (72)
VO_2_ (ml)	2205 (97)**	2198 (78)**	2229 (82)**	1491 (66)	1452 (63)	1483 (68)
HR (bpm)	149 (3)**	149 (2)**	150 (2)**	113 (2)	117 (2)	114 (2)
RER	0.99 (0.01)**	0.99 (0.01)**	0.99 (0.01)**	0.95 (0.01)	0.96 (0.01)	0.94 (0.01)
EqCO_2_	31.1 (0.6)**	30.6 (0.5)**	30.8 (06)**	30.8 (0.4)	29.6 (0.3)	29.9 (0.4)
EqO_2_	30.9 (0.8)**	30.5 (0.6)**	30.5 (0.7)**	29.2 (0.5)	28.5 (0.4)	28.1 (0.5)
V_E_ (l/min)	68.6 (3.7)**	67.3 (3.0)**	68.6 (3.5)**	43.7 (2.3)	41.7 (2.2)	41.8 (2.3)

SD are shown between parentheses. *Note*. VO_2_ = oxygen consumption; VCO_2_ = expired CO_2_; V_E_ = ventilation per minute; EqO_2_ = VO_2_/V_E_; EqCO_2_ = VCO_2_/V_E_; RER = respiratory exchange ratio. **p < 0.001 from Light Exercise condition.

### ANT-I RT

The typical main effects and interactions, usually observed with ANT-I task, for RT measures can be found as Supplementary Material S1. This pattern of results demonstrated the correct functioning of the task and therefore its suitability to assess the functioning of the classic attentional networks (see Table 3).

**Table 3:** Mean correct RT (ms) and Error Percentages for the experimental conditions of the ANT-I trials.

		Reaction Time		Error Percentages	
		Congruent	Incongruent	Congruent	Incongruent
No Tone	Invalid	514 (14)	560 (14)	2.03 (0.32)	3.50 (0.55)
	No Cue	523 (13)	555 (13)	3.07 (0.32)	3.44 (0.5)
	Valid	498 (13)	528 (12)	3.05 (0.36)	3.91 (0.59)
Tone	Invalid	508 (12)	561 (14)	1.10 (0.27)	2.58 (0.64)
	No Cue	498 (13)	539 (13)	0.9 (0.19)	1.59 (0.34)
	Valid	495 (14)	522 (14)	1.88 (0.39)	2.03 (0.32)

SD are shown between parentheses.

Importantly, there was a main effect of Exercise [*F* (1, 23) = 4.89, *p* = 0.037, ${\eta }_{p}^{2}$ = 0.18], with faster RT in the Moderate (*M* = 517 ms; *SD* = 12 ms) compared to the Light exercise (*M* = 535 ms; *SD* = 14 ms) condition. However, neither the main effect of Treatment nor the Exercise × Treatment interaction were significant (both *Fs* < 1). A detailed description of the modulations of Exercise and Treatment on the attentional networks’ indexes can be found in the Supplementary Material S1.

### ANT-I Percentage of errors

The typical main effects and interactions (see Table 4), usually observed with ANT-I task, for the errors rate performance can be also found in the Supplementary Material S1. The main effects of Exercise, Treatment, and the interaction Exercise × Treatment were not significant (all *F*s < 1, *p*s > 0.05), thus demonstrating that participants respond faster to ANT-I trials during ME, but without committing more errors. The modulations of Exercise intensity and Treatment on the attentional networks’ indexes are reported in the Supplementary Material S1.

**Table 4:** Mean correct RT (ms) and Error Rate (percentage of errors) as a function of the treatment and exercise conditions.

	Moderate Exercise			Light Exercise		
	Caffeine	Control	Placebo	Caffeine	Control	Placebo
**Reaction Time**						
Phasic Alertness	22 (5)	25 (5)	24 (5)	20 (5)	24 (5)	28 (6)
Orienting	23 (3)	19 (4)	27 (3)	25 (4)	26 (3)	29 (5)
Executive Control	34 (5)	42 (5)	40 (5)	35 (6)	35 (5)	35 (5)
**Error Rate**						
Phasic Alertness	1.58 (0.65)	2.30 (0.53)	1.50 (0.72)	0.35 (0.53)^*^	2.01 (0.44)	2.92 (0.60)
Orienting	−0.60 (0.54)	−0.61 (0.34)	−0.05 (0.46)	−0.48 (0.49)	0.16 (0.51)	−0.61 (0.60)
Executive Control	1.56 (0.60)	0.73 (0.49)	0.99 (0.46)	0.38 (0.62)	0.73 (0.47)	0.60 (0.55)

*Note*. *p = 0.012 from Control and Placebo in the Light Exercise condition.

### Executive vigilance

#### Mean RT

**Figure 2:**
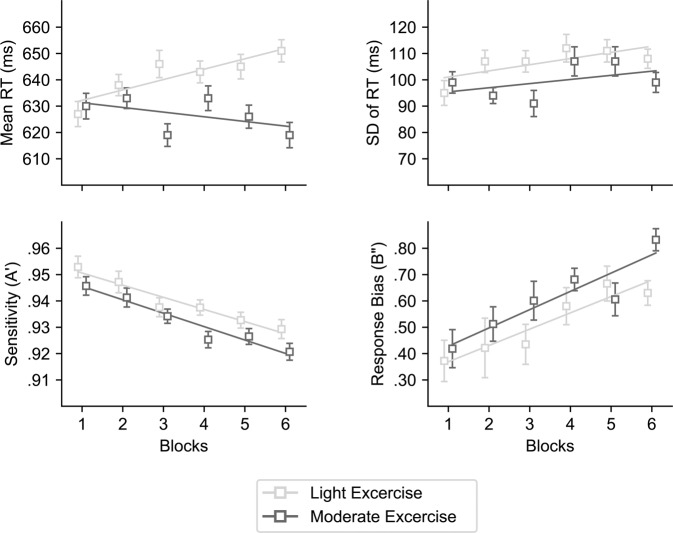
Executive Vigilance decrement as a function of exercise intensity (light or moderate). The solid lines represents the linear trend for each exercise condition across experimental blocks. Error bars represent standard error of the mean, calculated following Cousineau’s method for eliminating between participants variability^75^.

A main effect of Exercise was found close to the significance level [*F* (1, 23) = 3.99, *p* = 0.058, ${\eta }_{p}^{2}$ = 0.15], with faster RT during the Moderate (*M* = 627 ms; *SD* = 16.03 ms) than the Light exercise (*M* = 641 ms; *SD* = 16 ms) condition. However, the main effects of Treatment (*F* = 1.28, *p* > 0.05) or Blocks (*F* < 1) were not significant. Importantly, a significant interaction between Exercise and Blocks was observed [*F* (5, 115) = 3.73, *p* = 0.004, ${\eta }_{p}^{2}$ = 0.14]. The inspection of this interaction revealed that the linear component of mean RT across blocks was significantly different (*F* (1, 23) *=* 6.29; *p* = 0.020) for the Moderate vs. the Light exercise condition. A significant linear increment in mean RT was observed in the Light (*F* (1, 23) *=* 8.81, *p* = 0.010), but not in the Moderate exercise condition (*F* (1, 23) *=* 1.14, *p* = 0.30) (see Fig. 2). In other words, the main effect of exercise, with faster RT for the Moderate than the Light exercise condition, increased across blocks of trials. Last, no interaction for Treatment was observed as significant: Exercise × Treatment (*F* < 1), Treatment × Blocks (*F* = 1.00, *p* = 0.440) or Exercise × Treatment × Blocks (*F* = 1.05, *p* = 0.399).

#### SD of RT

The main effects of Exercise [*F* (1,22) = 1.00, *p* = 0.329, ${\eta }_{p}^{2}$ = 0.04], Treatment (*F* < 1, *p* > 0.05), or Blocks [*F* (5, 110) = 1.78, *p* = 1.23, ${\eta }_{p}^{2}$ = 0.07] were not significant. Also, no significant interactions were found for Exercise × Treatment (*F* < 1.00, *p* > 0.05), Exercise × Block [*F* (5, 110) = 1.24, *p* = *0*.295, ${\eta }_{p}^{2}$ = 0.05], Treatment × Blocks or Exercise × Treatment × Blocks (both *F*s < 1.00, *p*s > 0.05).

#### Sensitivity

Analysis didn’t show a main effect of Exercise [*F* (1, 23) *=* 2.72, *p* = 0.012, ${\eta }_{p}^{2}$ = 0.11] or Treatment (*F* = 0.73, *p* > 0.05), but did revealed a significant main effect of Blocks [*F* (5, 115) = 10.59, *p* < 0.001, ${\eta }_{p}^{2}$ = 0.32] with a linear decrement across time on task [*F* (1, 23) = 23.50, *p* < 0.001]. No interaction approached significance levels (all *ps* > 0.10).

#### Response Bias

Data showed no main effects of Exercise [*F* (1, 23) = 3.63, *p* = 0.070, ${\eta }_{p}^{2}$ = 0.14] or Treatment [*F* (2, 46) *=* 1.55, *p* = 0.223, ${\eta }_{p}^{2}$ = 0.03). However, there was a significant main effect of Blocks [*F* (5, 115) = 4.18, *p* = 0.001, ${\eta }_{p}^{2}$ = 0.15], which demonstrated a progressive linear increment across time on task [*F* (1, 23) = 21.85, *p* < 0.001, ${\eta }_{p}^{2}$ = 0.48]. No interaction approached significance levels (all *ps* > 0.363).

### Arousal vigilance

#### Mean RT

The main effect of Exercise was not significant (*F* < 1, *p* < 0.806). However, main effects of Treatment [*F* (1, 23) = 8.66, *p* = 0.001, ${\eta }_{p}^{2}$ = 0.27] and Blocks [*F* (5, 115) = 7.66, *p* < 0.001, ${\eta }_{p}^{2}$ = 0.25] were observed as significant. The Exercise × Treatment (*F* < 1), Exercise × Blocks [*F* (5, 115) = 1.46, *p* > 0.05, ${\eta }_{p}^{2}$ = 0.06], Treatment × Blocks [*F* (10, 230) = 1.40, *p* > 0.05, ${\eta }_{p}^{2}$ = 0.06] or Exercise × Treatment × Blocks interactions [*F* (10, 230) = 1.09, *p* > 0.05, ${\eta }_{p}^{2}$ = 0.05] were not significant.

**Figure 3:**
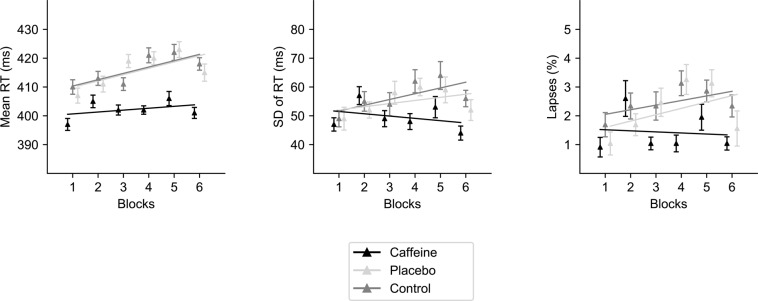
Arousal Vigilance decrement as a function of treatment conditions (5 mg/kg of caffeine intake, placebo, or control). The solid lines represent the linear trend for each treatment condition across the experimental blocks. Error bars represent standard error of the mean, calculated following Cousineau’s method for eliminating between participants variability^75^.

Planned contrasts for Treatment showed a significant faster mean RT in the Caffeine (*M* = 402 ms; *SD* = 6) compared with both the Control (*M* = 416 ms; *SD* = 7 ms) [*F* (1, 23*)* = 11.35, *p* = 0.003, ${\eta }_{p}^{2}$ = 0.33] and the Placebo (*M* = 416 ms; *SD* = 7 ms) [*F* (1, 23) = 10.59, *p* = 0.003, ${\eta }_{p}^{2}$ = 0.31] conditions, with no differences between Control and Placebo conditions (*F* < 1). Interestingly, note that RT increased linearly across blocks [*F* (1, 23) *=* 13.65, *p* = 0.001, ${\eta }_{p}^{2}$ = 0.37] until block 5^th^, but decreased in block 6^th^, as shown by the significant quadratic component [*F* (1, 23) = 8.17, *p* = 0.009, ${\eta }_{p}^{2}$ = 0.26] (see Fig. 3).

#### SD of RT

**Figure 4:**
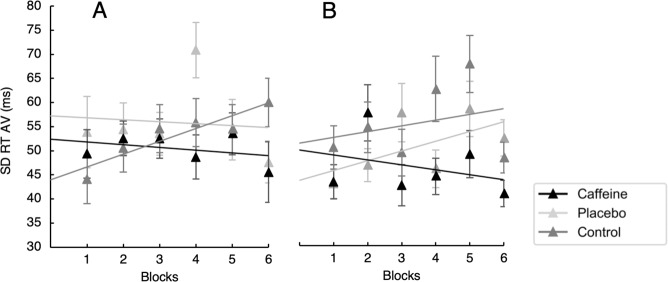
Illustration of the Exercise by Treatment by Block three-way interaction on SD of Arousal Vigilance (SD RT AV; in milliseconds). Data by experimental block and treatment condition in (**A**) Light intensity and (**B**) Moderate intensity exercise. Error bars represent standard error of the mean, calculated following Cousineau’s method for eliminating between participants variability^75^.

The main effect of Exercise was not significant (*F* < 1, *p* = 0.649), but it was marginal for Treatment [*F* (2, 46) = 3.06, *p* = 0.056, ${\eta }_{p}^{2}=\,0.12$] and significant for Blocks [*F* (5, 115) = 3.09, *p* = 0.012, ${\eta }_{p}^{2}$ = 0.12]. RT variability increased linearly up to block 5^th^, and decreased in block 6^th^, as shown by the significant quadratic component [*F* (1, 23) = 7.02, *p* = 0.013, ${\eta }_{p}^{2}$ = 0.23]. The effect of caffeine intake was moderated by the significant three-way interaction [*F* (10, 230) = 2.14, *p* = 0.023, ${\eta }_{p}^{2}$ = 0.08]. In particular, RT variability increased linearly in the Control condition in both the Light [*F* (1, 23) = 9.20, *p* = 0.006, ${\eta }_{p}^{2}$ = 0.29] and the Moderate exercise condition [*F* (1, 23) = 5.47, *p* = 0.028, ${\eta }_{p}^{2}$ = 0.19]. However, in the Placebo condition the increase was only observed in the Moderate exercise condition [*F* (1, 23) = 4.47, *p* = 0.046, ${\eta }_{p}^{2}$ = 0.16; F < 1 for the Light exercise condition]; whereas in the Caffeine condition RT variability did not change neither in the Moderate [*F* (1, 23) = 2.06, *p* = 0.164, ${\eta }_{p}^{2}$ = 0.08] nor in the Light exercise (*F* < 1) condition (see Fig. 4).

### Percentage of lapses

The main effect of Exercise was not significant (F < 1), but it was marginal for Treatment [*F* (2, 46) = 2.62, *p* = 0.078, ${\eta }_{p}^{2}$ = 0.10], and significant for Blocks [*F* (5, 115) = 4.11, *p* = 0.002, ${\eta }_{p}^{2}$ = 0.15]. Lapses also showed an initial linear increase up to block 5^th^, and a decrease in the block 6^th^, statistically supported by the linear [*F* (1, 23) = 3.26, *p* = 0.084, ${\eta }_{p}^{2}$ = 0.12] and quadratic [*F* (1, 23) = 9.61, *p* = 0.005, ${\eta }_{p}^{2}$ = 0.29] components.

Moreover, the Exercise × Treatment interaction was significant [*F* (2, 46) = 3.37, *p* = 0.043, ${\eta }_{p}^{2}$ = 0.13], but not the Exercise × Block, Treatment × Block or Treatment × Exercise × Block interactions (all *F*s < 1, *p*s > 0.05). Partial ANOVAs on each level of Exercise showed that the main effect of Treatment was only significant in the Moderate exercise condition [*F* (2, 46) = 6.70, *p* = 0.003, ${\eta }_{p}^{2}$ = 0.23], with a reduced percentage of lapses in the Caffeine (1.13%) compared to the Control (2.91%) and Placebo (2.17%) conditions. Instead, no significant main effect of Treatment was observed in the Light exercise condition (*F* < 1).

## Discussion

The present study aimed at assessing the effects of caffeine intake during exercise, mainly on executive and arousal vigilance, but also in the classic attentional networks functions. To do so, two different exercise intensities were used to compare the impact of acute exercise and caffeine intake. Behavioral performance was assessed with a task (the ANTI-Vea) suitable to measure in a single session phasic alertness, orienting, and executive control functions, along with the decrement of two vigilance components (arousal and executive vigilance) across time on task^4^. To our knowledge, this is the first study assessing the effects of exercise and caffeine on executive and arousal vigilance in the same experimental session, providing new additional information about the effects of 5 mg/kg caffeine ingestion on the functioning of the attentional networks during exercise.

Our results suggest that moderate exercise prevent an executive vigilance decrement (but not arousal vigilance) during a ~30 min physical effort. On the contrary, while caffeine ingestion seems to have no effect on executive vigilance during exercise, it improved arousal vigilance compared to the placebo and control treatments, reducing reaction time and interacting with moderate exercise to reduce the number of lapses committed across time on task. It is worth mentioning that the reaction time modulation was independent of exercise intensity, suggesting that caffeine ingestion would be capable of improving arousal vigilance despite the attentional benefit of moderate exercise, but also would particularly reduce lapses during the most challenging exercise condition.

Regarding the classic attentional networks, previous studies had shown faster overall responses at the expense of accuracy (known as tradeoff effect) during ANT-I tasks and moderate-to-high exercise intensities (e.g., 80–90% VT_2_), in comparison to a rest condition^31,32^. Complementary to these reports, our research found the same global RT facilitation effect, in this case compared to light exercise (80% VT_1_), but without any increase in error rate. Despite these differences, the absence of a tradeoff effect in our study seems to be in line with several studies suggesting that moderate exercise intensity may not be “stressful” enough to produce sympathetic-adrenal changes that impair error rate compared to lower intensities^47–49^.

Nevertheless, contrary to our hypothesis, no significant modulation of caffeine ingestion over global RT on the ANT-I sub-task was observed, at difference with previous studies^29,38,50^. Note, however, that Huertas *et al*.^31^ observed that caffeine ingestion facilitated RT at rest in moderate caffeine consumers, but not during moderate exercise. The basis for caffeine attentional effects relays on its role as an adenosine antagonist (blocking A_1_ and A_2a_ receptors, highly present in striatum, hippocampus, cortex, cerebellum, and hypothalamus brain regions) therefore promoting the release of neurotransmitters (e.g., dopamine, noradrenaline, acetylcholine or serotonin) and increasing arousal^24^. Still, following the Locus Coeruleus – Norepinephrine (LC-NE) hypothesis^51^, and as Huertas *et al*.^31^ emphasized, both exercise and caffeine intake would have equally stimulated the LC-NE system, explaining the absence of results in our study. Therefore, as in the present study caffeine treatment was always analyzed in conditions of at least light exercise, this could have caused an arousal ceiling effect of exercise, masking the effects of caffeine on the classic attentional networks. Regarding error rates, our results are in line with previous research^29,31^ in which it has been shown that after caffeine ingestion (150–450 mg), either an extremely low error rate or even improved performance is observed in complex attentional tasks^24^.

Regarding arousal vigilance, we observed a decrement in arousal vigilance across time on task, with lapses increasing over time independently of exercise intensity, as in previous research at rest^4^ and during exercise^19^. Rationale for this behavioral pattern seems to follow the overload hypothesis^52^: cognitive resources were progressively depleted during a high demanding cognitive task (e.g., the ANTI-Vea) and physical effort (light and moderate), impairing attention and increasing lapses. However, this result implied that the moderate exercise condition had no effect on arousal vigilance, which was one of our main hypotheses. Limited research had previously shown a reduction in mean RT by light-to-moderate exercise (40–80% VT_2_), but also conflicting results during high intensities, and no effect on vigilance decrement^17,18^.

Arousal vigilance has not been so far assessed specifically along with other attentional networks in a complex task, being a factor that influences vigilance performance^33^. Therefore, the complexity of the ANTI-Vea task, due to the simultaneous assessment of the executive vigilance component (and the other attentional functions), at difference with previous research only using the PVT^53^, could explain why, contrary to our initial hypothesis and also to Gonzalez-Fernández *et al*. work^18^, moderate exercise (80% VT_2_) intensity produced no beneficial effect compared with light exercise (80% VT_1_). Altogether, the results by Gonzalez-Fernández *et al*.^18^ seems to be in line with the reduction on executive vigilance decrement by the moderate exercise intensity observed in our experiment, that will be discussed latter. Nevertheless, our work could complement Huertas *et al*.^32^ proposal, suggesting that vigilance improvement may only occur when comparing rest conditions with moderate exercise, and not between two exercise intensities due to the activation induced by the minimal exercise.

On the contrary, arousal vigilance was modulated by caffeine ingestion, with 5 mg·kg of caffeine leading to faster responses and fewer lapses during moderate exercise intensity. These results provide new insights into caffeine effects on arousal vigilance during a concomitant physical effort. Our results agree with McLellan *et al*.^28^ work, who described a reduction in the number of lapses committed during a military specific vigilance exercise after a 200 mg caffeine intake, and with the solid body of evidence in resting condition, with or without sleep deprivation^22,54,55^. In this regard, it is well known that caffeine blocks vigilance related adenosine receptors in the brain (e.g., hippocampus, cortex, cerebellum) causing a general effect of vigilance^56^. Additionally, the placebo ingestion during light exercise conditions (not in moderate intensity) mildly reduced RT variability, which supports placebo’s significant cognitive^57^ and psychomotor^58^ effects through the release of dopamine in prefrontal cortex, but highlights that it could be limited to low activation states.

Regarding executive vigilance, we expected the decrement across time on task on this component to be observed as a change in response bias towards committing fewer errors. This would had major implications in a real situation, as the tendency to be more “conservative” during a critical and unexpected event could lead, for example, to confound a quick turn to avoid a crash of a cyclist with a normal movement of the peloton during a cycling ride (e.g., the cyclist has to see the danger very clearly to respond to it). In this regard, while the great majority of scientific studies haven’t found significant changes in response bias over time (mostly due to limited task complexity^7^), our data showed, partially confirming our hypothesis, a significant reduction in hits accompanied by a reduction in sensitivity and a significant increase in response bias across blocks, independent of exercise intensity. These results differ from data collected by Luna *et al*.^4^ at rest, and contrast with Thomson *et al*.^7^ hypothesis. To explain these results, we suggest that dual task demands in our study exaggerate the executive vigilance decrement due to the reallocation of cognitive resources^59^, allowing us to see both a sensitivity loss and an increase in response bias during the ANTI-Vea, not observed by Luna *et al*.^4^.

Indeed, other studies assessing the executive component during exercise have also found a decrement of vigilance performance with time on task^15,29^, that might be magnified when heavy loaded physical efforts are performed^15^. Still, based on neuroelectric findings with light intensity efforts (improved attentional resource allocation: increased α1 and β1 power measures of an electroencephalogram)^13^ and exercise-related norepinephrine release, we hypothesized that higher exercise intensities would enhance executive vigilance, and possibly reduce vigilance decrement. We observed that moderate exercise intensity prevented the increase in RT observed during light exercise, thus avoiding a significant vigilance decrement. Although this result opposes a reported increase in vigilance decrement during “loaded walking” vs. “unloaded walking” with a Go/NoGo task^15^, physical effort (120 min of complex, heavy loaded and variable inclination walking vs. constant light and moderate aerobic exercise in a cyclergometer) could explain the differences with our vigilance outcomes.

Moreover, our results agree with one report of improved brain activation, but no behavioral performance changes, in a SART task after light exercise (120–150 heart beats per minute) compared to rest^60^ and support the role of LC-NE^51^, possibly explaining exercise modulation of executive vigilance through a better maintenance of excitation during the ANTI-Vea task. Following the LC-NE hypothesis, maintenance of excitation throughout a vigilance task depends on tonic activation of the neurons associated to dorsolateral pre-frontal cortex, frontal eye fields, intraparietal sulcus, thalamus and anterior cingulate cortices^46,61^; and any decrease in their activation frequency would lead to the decrease of performance observed in vigilance tasks^62^. Indeed, it has been recently shown that stimulation with transcranial direct current stimulation over the posterior parietal or the dorsolateral pre-frontal cortex reduces the executive vigilance decrement^63^. In this scenario, low-to-moderate exercise would stimulate the synthesis and release of norepinephrine from the locus coeruleus to the rest of the brain, thus maintaining tonic activation and preventing performance decrease^14^.

Therefore, based on Smit *et al*.^13^ work and our results, a moderate intensity exercise could be necessary (e.g., 80% VT_2_) to induce a sufficient increase in catecholamines to activate and maintain executive vigilance related brain areas and observe a behavioral change. Nevertheless, our findings suggest that intensity selection can be crucial in a wide range of sport modalities that aim at maintaining executive control during an exercise period of 30–35 min. In these particular cases, a moderate intensity effort can contribute to reduce executive vigilance decrements and enhance global attentional performance. However, future research should confirm this hypothesis in specific sport contexts (e.g., outside the lab).

Lastly, regarding caffeine, no significant modulation of executive vigilance by caffeine or placebo ingestion, neither an interaction between caffeine ingestion and exercise, was observed. These results contradict available evidence regarding caffeine effects on vigilance during^29,38^ or after exercise^27^, but nevertheless are in line with Shulder *et al*.’^16^ study. The lack of measures during the first 30 min of exercise^29^, caffeine dosage^29,38^, but mostly the attentional task and participant characteristics could explain this controversy. One possible explanation is that low prevalence of infrequent signals in previous studies^29,38^ (10% vs. 20% in our task), as well as the fact that the ANTI-Vea demands responses to both frequent and infrequent stimuli, may have influenced the effects of caffeine. A second, and perhaps more solid explanation, is that habitual caffeine intake could influence acute caffeine effects on executive vigilance, as suggested for the effect on the classic attentional networks^31^. In this regard, while Shulder *et al*.^16^ and our sample were low (<100 mg/day) or non-habitual (<50 mg/day) caffeine consumers, participants habitually consumed 100–225 mg/day in studies that have found a positive impact of caffeine ingestion on executive vigilance, during or after exercise^27,29,38^.

Still, the absence of a significant interaction between exercise and caffeine on executive vigilance and opposed caffeine effects on executive and arousal vigilance remain to be explained. Following information processing theories, a psychomotor dual-task would reduce the amount of cognitive resources allocated to the cognitive task, impairing its performance, but caffeine has been proposed to counteract this reduction with an increase of brain activity^38^. From this, one would expect higher caffeine effects on vigilance as physical demands of dual task increases (e.g., increasing exercise intensity from light to moderate). Since our work is the first to analyze the interaction between caffeine and sub-maximal physical exercise in executive vigilance, it is difficult to draw solid conclusions, but, in order to explain the absence of caffeine-exercise interaction, it is likely that the two vigilance components trigger the brain differently, both on location and intensity, determining caffeine effects. Arousal vigilance has been associated to right hemisphere activation^64,65^, while there is evidence of how activation of the left hemisphere (e.g., lower frontal gyrus) is critical for inhibition of motor response (a critical ability in executive vigilance tasks^66^). As caffeine appears to increase brain activity, especially in the right hemisphere^67^, we suggest that executive vigilance could be less responsive to caffeine effects. Nevertheless, this hypothesis needs to be proven in future research with neuro-physiological measures like fMRI, fNIRS or EEG. The fact that an on-line version of the ANTI-Vea task is freely available (https://www.ugr.es/~neurocog/ANTI/) in different languages might help to extend research in this field with consistent measures in different studies.

Finally, we expected caffeine intake to reduce phasic alertness in low consumers during exercise and explore the effects of caffeine and exercise over the functioning of the attentional networks, extending previous research with ANT and ANT-I tasks. In this sense, caffeine has been shown to improve neuronal activity and neurotransmitters release^68^, thus improving phasic alertness^69^, executive control^70^ and orienting^71,72^ at rest, in a similar way to exercise arousal effects through catecholamine’s increase and dopaminergic system stimulation^73^. As happened with global RT, exercise influence on dopaminergic system would have caused a ceiling effect and masked the effects of caffeine on the RT phasic alertness index. Still, we did find a significant improvement in the error rate phasic alertness index after caffeine ingestion, that was only present in light exercise condition and agrees with a superior increase of plasma catecholamines during moderate intensity exercise^74^.

### Limitations

Several limitations of the study are worth noting. Firstly, individual caffeine assimilation was not assessed. Secondly, time of day was not controlled as an independent variable. Therefore, circadian rhythm might have influenced physiological outcomes. Future studies should overcome these limitations.

## Conclusions

To conclude, our results reveal that executive and arousal vigilance are affected differently by caffeine and exercise. While caffeine improved arousal vigilance during light and moderate exercise, additionally reducing lapses committed during moderate exercise, it had no effect on executive vigilance. Quite the reverse, moderate exercise improved reaction time on executive vigilance, while arousal vigilance and the attentional networks functions remained unaffected by the exercise manipulation. Additionally, supporting previous theorizing^7^, our results show that vigilance decrement during aerobic exercise also occurs mainly due to a change in response strategy towards more conservative responses, rather than by a loss of sensitivity across time on task.

## Supplementary information

Supplementary material 1.

## Data Availability

The dataset generated and analyzed during the current study is available in the OSF repository, https://osf.io/kbypa/.
