# Supplementary material for: Effects of caffeine intake and exercise intensity on executive and arousal vigilance
Source: Sci Rep. 2020 May 21;10:8393. doi: 10.1038/s41598-020-65197-5 (PMC7242431; doi:10.1038/s41598-020-65197-5)
Supplement: Supplementary file 1 — Supplementary material 1. [file 41598_2020_65197_MOESM1_ESM.docx]

**Effects of caffeine intake and exercise intensity on executive and arousal vigilance**

Supplementary Material

Authors: Carlos Sanchis^1,*^, Esther Blasco^1^, Fernando G. Luna^2,3,+^, Juan Lupiáñez^2,+,*^

Affiliations:

^1^ Catholic University of Valencia, Valencia, 46001, Spain

^2^ University of Granada, 18071, Granada, Spain

^3^ Universidad Nacional de Córdoba, Córdoba, Argentina

Corresponding authors:

* [carlos.sanchis@ucv.es](mailto:carlos.sanchis@ucv.es)

[* jlupiane@ugr.es](mailto:*%20jlupiane@ugr.es%20)

**Results**

## ANT-I RT

The typical effects and interactions, usually observed with ANT-I task, were found for Phasic Alertness [*F* (1, 23) = 11.78, *p* = .002, $\eta_{p}^{2}$ = .34], Orienting [*F* (2, 46) = 61.82, *p* < .001, $\eta_{p}^{2}$ = .73], Executive Control [*F* (1, 23) = 76.91, *p* < .001, $\eta_{p}^{2}$ = .77], Phasic Alertness × Orienting [*F* (2, 46) = 24.79, *p* < .001, $\eta_{p}^{2}$ = .52], Phasic Alertness × Executive Control [*F* (1, 23) = 12.73, *p* = .002, $\eta_{p}^{2}$ = .36], Orienting × Executive Control [*F* (2, 46) = 21.24, *p* < .001, $\eta_{p}^{2}$ = .48], and Phasic Alertness × Orienting × Executive Control [*F* (2, 46) = 7.84, *p* = .001, $\eta_{p}^{2}$ = .25].

For Phasic Alertness, responses were faster in the Tone (*M =* 521 ms; SD = 13) than in the No Tone condition (*M =* 531 ms; SD = 12). For Orienting, the typical orienting effect was observed, with faster responses for Valid trials (*M =* 511 ms; SD = 13), than for No Cue (*M =* 530 ms; SD = 13) and Invalid trials (*M =* 536 ms; SD = 13). Last, for Executive Control, the interference effect was found with faster RT for Congruent (*M =* 507 ms; SD = 13) than for Incongruent trials (*M =* 544 ms; SD = 13).

Furthermore, regarding the modulations of Exercise intensity and Treatment on the attentional networks’ indexes, no main effects of Exercise, Treatment or Exercise × Treatment interaction were found for the Phasic Alertness index (all *Fs <* 1). Similarly, no main effects of Exercise (*F =* 3.10, *p =* .09), Treatment (*F =* 1.11, *p =* 0.34), or Exercise × Treatment interaction (*F <* 1) were found on the Orienting index. Likewise, no main effects of Exercise (*F =* 1.94, *p =* .18), Treatment (*F =* 1.04, *p =* 0.36) or interaction of Exercise × Treatment (*F =* 1.40, *p =* .26) on Executive Control index reached statistical significance (see Table 4).

## ANT-I Percentage of Errors

ANOVAs for percentage of errors reproduced the ANT-I typical effects, with a main effect of Phasic Alertness [*F* (1, 23) = 38.63, *p* < .001, $\eta_{p}^{2}$ = .63], where % of errors were reduced in the Tone (*M =* 1.68 %; *SD* = 0.21 %) compared to the No Tone condition (*M =* 3.16 %; *SD* = 0.34 %) trials. As expected in the ANT-Vea task, Orienting [*F* (2, 46) = 2.59, *p* =.086, $\eta_{p}^{2}$ = .10] and Executive Control [*F* (1, 23) = 4.00, *p* = .057, $\eta_{p}^{2}$ = .15] effects did not reach statistical significance.

Regarding the attentional indexes, the corresponding repeated measures ANOVAs showed no effect of Exercise on Phasic Alertness (*F* < 1), Orienting (*F =* 1.81, *p =* .175) or Executive Control (*F =* 1.41, *p =* .247). Nevertheless, a main effect of Treatment [*F* (1, 46) = 3.83, *p* = .029, $\eta_{p}^{2}$ = .14] on Phasic Alertness, but not on Orienting (*F =* 0.06, *p >* .05) or Executive Control (*F =* 1.40, *p >* .05), was found. No Exercise × Treatment interaction was found for Phasic Alertness (*F* = 1.58, *p =* .216), Orienting (*F* < 1) or Executive Control (*F =* 1.10, *p =* .34).
